# Transmembrane Pressure during Micro- and Diafiltration of Milk Affects the Release of Non-Sedimentable Caseins

**DOI:** 10.3390/foods12112234

**Published:** 2023-06-01

**Authors:** Norbert Raak, Özgenur Coşkun, Milena Corredig

**Affiliations:** 1 Department of Food Science, Aarhus University, Agro Food Park 48, 8200 Aarhus N, Denmark; ozgenurcoskun@food.au.dk (Ö.C.); mc@food.au.dk (M.C.); 2 CiFOOD Centre for Innovative Food Research, Aarhus University, 8000 Aarhus, Denmark

**Keywords:** membrane filtration, microfiltration, casein micelles, milk protein concentrate, transmembrane pressure, whey protein depletion

## Abstract

Membrane filtration, especially in combination with diafiltration, can affect the colloidal structure of casein micelles in milk and concentrated milks. The partial dissociation of casein proteins from the casein micelles into the serum phase has been shown to depend on diafiltration conditions. This dissociation can affect the technological functionality of the milk concentrates. The present study aimed at determining the contribution of the gel layer deposited onto the membrane during filtration in the colloidal equilibrium between soluble and micellar caseins. Skimmed milk was concentrated by microfiltration combined with diafiltration using a cross-flow spiral-wound membrane at two transmembrane pressure (TMP) levels, causing differences in the extent of the gel layer formed. Non-sedimentable casein aggregates were formed to a greater extent at a low TMP compared to a high operating TMP. This difference was attributed to the greater compression of the deposit layer during filtration at a high TMP. This study contributes new knowledge to the understanding of how to modulate the functionality of milk concentrates through the control of processing conditions.

## 1. Introduction

Membrane filtration is a fractionation technology often utilised in dairy processing to obtain functional ingredients. In microfiltration, skimmed milk is passed over a membrane layer with pores large enough to allow for the transmission of whey proteins and small molecular weight constituents, which are typically 0.08–0.2 µm, to increase the casein micelle fraction. This process is often combined with diafiltration, which is the introduction of water or other media to enhance the transmission of permeable compounds while maintaining high efficiencies [1]. Microfiltration in combination with diafiltration is a common way to produce micellar casein concentrates (MCCs), i.e., milk protein concentrates with an increased ratio of casein to whey proteins [2,3].

For several years, the microfiltration of milk, with or without diafiltration, has been studied in detail from both the processing and product side. The permeate throughput per membrane area and time, referred to as flux, and whey protein permeability are the main target process parameters that need to be optimised by identifying the optimal conditions for, e.g., feed concentration, nominal pore size, and transmembrane pressure (TMP). The latter is the pressure gradient between the retentate and the permeate side. The permeate flux increases with TMP until it reaches a plateau, referred to as the limiting flux, where the formation of a deposit layer on the membrane with increasing TMP counteracts the permeation [4]. When the concentration factor of the feed increases, the limiting flux decreases and is reached at a lower TMP [5,6,7]. In addition, the composition of the permeate may change with TMP due to the formation of the deposit layer. For example, it has been shown that whey protein permeation decreases with increasing TMP once above the limiting flux [8,9,10], as the specific resistance of the deposit layer increases and its porosity decreases with increasing TMP [10,11]. The deposit layer was moreover shown to be composed of both casein micelles and the whey protein β-lactoglobulin [12], meaning a further decrease in whey protein permeation due to attachment to the deposit layer. Diafiltration with water was shown to increase flux due to a reduction in feed viscosity via the removal of lactose, minerals, and proteins [6,7]. However, the diafiltration medium has an effect. For example, lower fluxes were reached as the hardness of the diafiltration water was increased [13]. Continuous diafiltration while keeping the feed volume constant can achieve better flow than discontinuous batch diafiltration [14]. However, this may not correspond to efficiencies in whey protein permeation: while continuous diafiltration at a constant feed volume resulted in the highest flux, the highest whey protein permeation was achieved using continuous diafiltration with simultaneous volume reduction or intermittent diafiltration [14].

The microfiltration of milk is usually performed at either 10 or 50 °C. While at 50 °C it is possible to operate at high flow rates and whey proteins permeate efficiently through the membrane, the tendency towards fouling is lower and microbial quality better at a process temperature of 10 °C. However, as the feed viscosity is high, low temperatures result in lower flux values [8]. Additionally, β-casein is known to partly dissociate from the casein micelles at <10 °C, and its presence in the serum implies that it can be transmitted through the membrane and change the permeate composition.

Concentrating casein micelles by membrane filtration, especially in combination with diafiltration, affects the colloidal structure of casein micelles, which in turn, affects the techno-functional properties of the final concentrate [15]. In particular, the partial dissociation of casein molecules into the serum phase as a consequence of the milieu exchange and increased interactions between casein micelles have been reported [16,17,18]. The presence of non-sedimentable caseins in the serum phase can impart important differences onto the techno-functionality of the concentrates. For example, it can affect the rennet-induced aggregation of the casein micelles in the final product [19].

Thus far, little attention has been given to the impact of the deposit gel layer formed on the membrane on the physical and chemical characteristics of the resulting milk concentrate. It might be hypothesised that the presence of a deposit layer is related to a higher extent of casein dissociation from the micelles, as, during the filtration process, the shear forces are greatest close to the membrane surface and may drive the partial disruption of casein micelles from the deposit layer. This hypothesis was tested by processing a milk concentrate at two different TMPs. The aim of the present research was to study whether a difference in transmembrane pressure during the diafiltration of MCCs in cross-flow spiral-wound membranes can change the amount of non-sedimentable casein aggregates in the final retentate. This has never been investigated before, even though knowledge on the formation of non-sedimentable casein aggregates as affected by the processing conditions is crucial to predict the functionality of the resulting MCCs.

## 2. Materials and Methods

### 2.1. Sample Preparation

Pasteurised skimmed milk was obtained from Arla Foods amba (Viby, Denmark) and incubated in a water bath at ~50 °C for 30–60 min prior to the experiments. Microfiltration was performed using a modified pilot unit model R2, ID180334 (GEA Group AG, Düsseldorf, Germany), equipped with a ∅16 cm × 97 cm PVDF spiral-wound membrane module with a 0.1 µm cut-off and 17.1 m^2^ membrane area (V0.1-3B-6438; Synder Filtration, Vacaville, CA, USA). The water in the system was displaced with 80 L of skimmed milk before the experiment was started. A total of ~420 L of skimmed milk was continuously added to the process while constantly removing the permeate in ~30–50 L intervals and recirculating the retentate to concentrate the casein micelle fraction in the feed tank. During the concentration step, the transmembrane pressure (TMP) was kept constant at 0.05 MPa. After removing ~360 L of permeate, continuous diafiltration with ~100 L of soft water was conducted at a TMP of either 0.05 or 0.1 MPa. The water was added automatically by the system to maintain the level in the feed tank. The experiments were conducted at 50 °C. The feed–retentate ratio was set to 1.2, with a targeted retentate flow of 100 L h^−1^, and the circulation loop pump was run at 45% of the maximum capacity. Then, 0.2 g L^−1^ sodium azide was added to all withdrawn samples to prevent spoilage.

Skimmed milk and retentate samples were centrifuged at 100,000× *g* and 20 °C for 1 h (Optima L-80 XP; Beckman Coulter, Inc., Brea, CA, USA; rotor type Ti70) to obtain the serum phases for further investigations [16].

### 2.2. Protein Analysis

The protein contents of skimmed milk and retentates as well as their centrifugal supernatants were determined using a Gerhardt Dumatherm (C. Gerhardt GmbH & Co.KG, Königswinter, Germany) with a conversion factor of N × 6.38 [16].

Whey protein content of the permeates was analysed by reversed-phase high-performance liquid chromatography, as described previously in detail [16]. The concentrations of α-lactalbumin and β-lactoglobulin were calculated from the peak areas using calibration curves (0.1–40 mg L^−1^) obtained from commercial standards (Sigma-Aldrich, Steinheim, Germany).

Proteins in the centrifugal supernatants were analysed in native conditions by size exclusion chromatography (20 mmol/L Bis-Tris propane buffer; Sephacryl S-500 high-resolution resin, GE Healthcare, Uppsala, Sweden), as described previously [20], to separate the whey proteins and non-sedimentable casein aggregates.

### 2.3. Retentate Viscosity

The viscosities of the skimmed milk and microfiltration retentates were measured according to Coşkun et al. [16] with slight modifications, using a stress-controlled AR-G2 rheometer (TA Instruments, New Castle, DE, USA) equipped with a DIN concentric cylinder geometry ($d_{i}$ = 28 mm, $d_{o}$ = 30 mm, h = 42 mm) and a Peltier element for temperature control. A logarithmic shear ramp from $\dot{\gamma }$ = 10 to 300 s^−1^ was run for 120 s, and 10 points per decade were recorded. The measurement temperature was 50 °C (equal to the filtration temperature), and the samples were heated in a water bath at 50 °C for 10–30 min before the measurements. The apparent viscosity is reported at $\dot{\gamma }$ = 200 s^−1^.

### 2.4. Calcium and Phosphate Analysis

The calcium and phosphate contents of skimmed milk, microfiltration retentates and permeates, and centrifugal supernatants were determined using inductively coupled plasma mass spectrometry (ICP-MS 7900, Agilent Technologies, Inc., Santa Clara, CA, USA) as described previously [21]. Briefly, sample aliquots (20 µL for retentates, 100 µL for all other samples) were blended with 1 mL HNO_3_ in glass vials and digested at 1400 W (max. temperature 145 °C) in a microwave oven (Multiwave 3000, Anton Paar GmbH, Graz, Austria) equipped with a rotor (model 64MG5, Anton Paar GmbH). The digested samples were diluted with ultrapure water before injection for ICP-MS. The general-purpose plasma mode was used to quantify ^43^Ca and ^31^P isotopes at a sample depth of 129 mm and a nebuliser flow rate of 0.5 rps. Helium was used to reduce polyatomic interference, and yttrium was used as the internal standard.

### 2.5. Statistical Analysis

All analyses were performed twice; the results are reported as mean values. Statistical differences between milk protein concentrates diafiltered at low and high TMPs were evaluated by Welch’s *t*-test (p < 0.05).

## 3. Results

**Figure 1:**
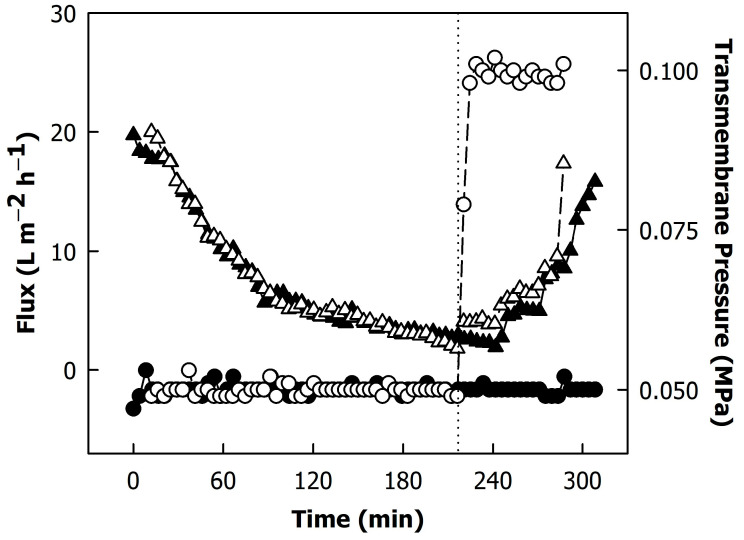
Flux (triangles) and transmembrane pressure (circles) during filtration of skimmed milk through a 0.1 µm spiral-wound PVDF membrane module in combination with diafiltration against soft water. The dotted line indicates the beginning of the continuous diafiltration step. Transmembrane pressure during diafiltration was 0.05 MPa (closed symbols) or 0.10 MPa (open symbols).

Figure 1 illustrates the flux (i.e., permeate flow per membrane area) and TMP during the filtration process. The flux decreased gradually over time with concentration. This is typically due to the gradual formation of a deposit layer on the membrane, causing a higher resistance to permeation [8]. Additionally, the continuous increase in feed viscosity with increasing concentration lowered the permeate flow, as previously demonstrated [6,7]. In the concentration step, the development of the flux with processing time was identical in both independent trials, demonstrating the reproducibility of the process. The continuous diafiltration with soft water (3.0 mg L^−1^ calcium and 0.3 mg L^−1^ phosphate) was initiated after removing ~360 L of permeate (Figure 1; dotted line), and the TMP was either kept at 0.05 MPa or increased to 0.1 MPa during this part of the process.

During diafiltration, the flux increased continuously because of a decrease in viscosity of the concentrates due to the milieu exchange [7]. At a higher TMP, there were no differences in the flux, indicating that 0.05 MPa was already close to the limiting flux for this process, which is in agreement with previous reports [5].

**Figure 2:**
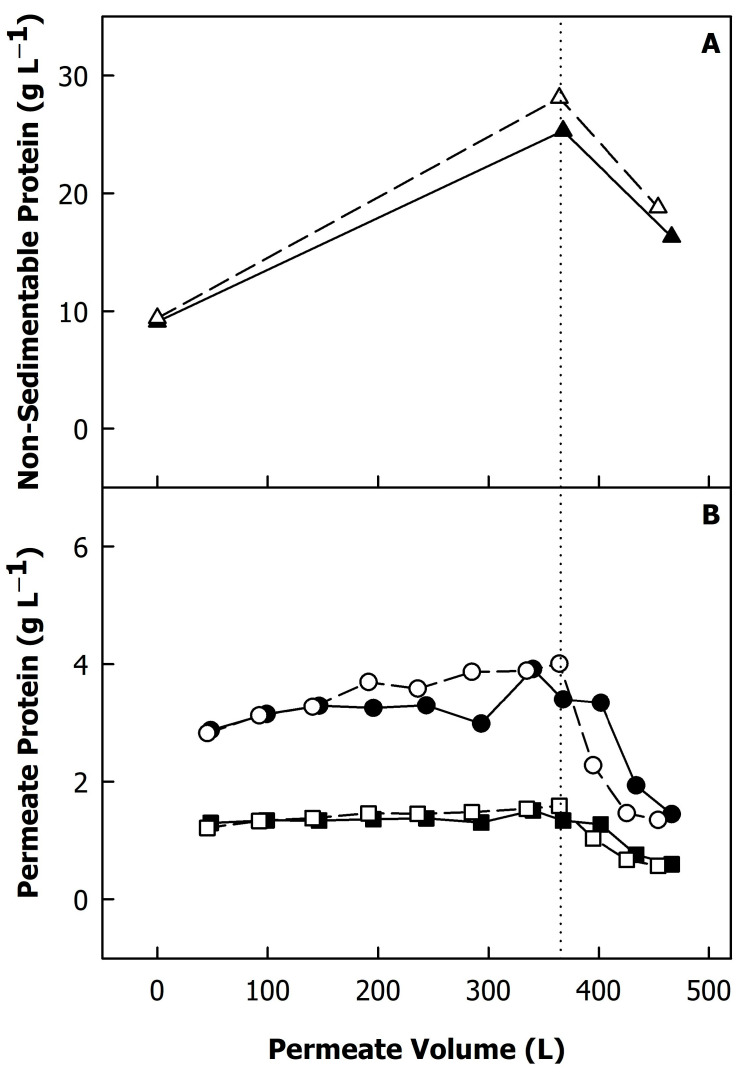
Non-sedimentable protein content of skimmed milk and milk protein concentrates during microfiltration and diafiltration (**A**), and α-lactalbumin (squares) and β-lactoglobulin concentration (circles) of the microfiltration permeates and different stages of the process (**B**). The dotted line indicates the beginning of the diafiltration step. Transmembrane pressure during diafiltration was 0.05 MPa (closed symbols) or 0.10 MPa (open symbols).

**Figure 3:**
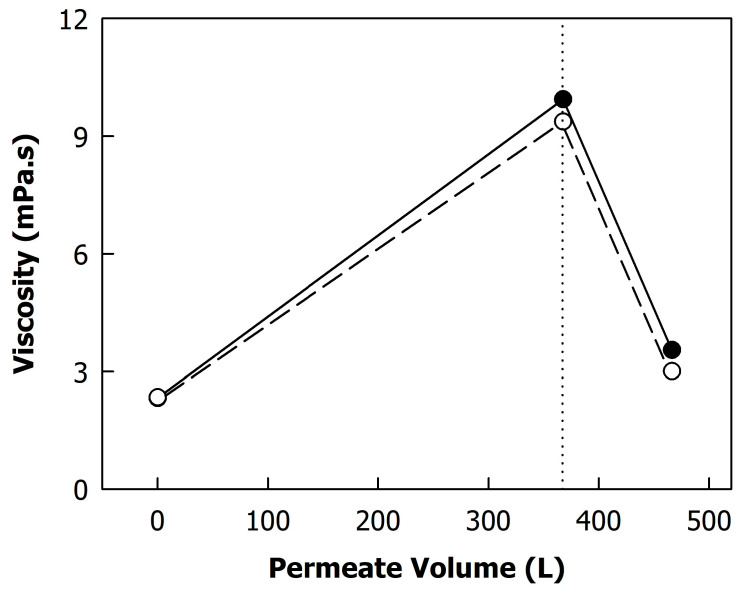
Viscosity of skimmed milk and milk protein concentrates during microfiltration and diafiltration. The dotted line indicates the beginning of the diafiltration step. Transmembrane pressure during diafiltration was 0.05 MPa (closed symbols) or 0.10 MPa (open symbols).

The total protein content increased from 34 g/L in skimmed milk to 176 g/L with concentration, corresponding to a concentration factor of ~5×. The concentration of non-sedimentable proteins (in the centrifugal supernatants) increased to a lesser extent due to the permeation of whey proteins (Figure 2A). Figure 2B shows the concentration of α-lactalbumin and β-lactoglobulin in the permeate samples collected at different time points of the process. The whey protein concentration in the permeates remained fairly constant during concentration. Due to the recirculation of the retentate, the whey proteins that did not pass the membrane were concentrated together with the casein micelles, leading to a higher concentration in the feed stream and thus a greater extent of permeation compared to a process where the feed concentration is kept constant [6]. Therefore, the whey protein concentration in the permeates decreased after the final concentration factor was reached and diafiltration started (Figure 2; dotted line). Differences between diafiltration at low and high TMPs were significant only for the first permeate sample (permeate volume of ~395 L), where a lower β-lactoglobulin content was found in the permeate obtained from diafiltration at a high TMP (p < 0.05). A lower extent of whey permeation at a higher TMP due to compression of the deposit layer was also reported by Hartinger et al. [8]. In contrast, France et al. [22] did not observe differences in whey protein permeation between filtration below and above the limiting flux. Diafiltration decreased the total protein content of both microfiltration retentates to ~120 g/L, which is a greater extent than expected from the amount of permeated whey proteins and concentration of serum proteins, indicating that casein was also transmitted through the membrane. In fact, it was found by RP-HPLC that casein was present in all permeate samples (data not shown). It was previously reported that a milieu exchange with soft water can result in the disintegration of casein micelles, especially at 50 °C [13]. This could explain the loss of some of the micellar caseins during the diafiltration step. Together with the protein content of the microfiltration retentates, their viscosity at 50 °C decreased by diafiltration from ~9.7 to ~3.3 mPa·s (Figure 3), which also explains the increase in the flux during diafiltration (Figure 1). The differences in protein content and viscosity between retentates obtained from diafiltration at low and high TMPs were not significant (p > 0.05).

**Figure 4:**
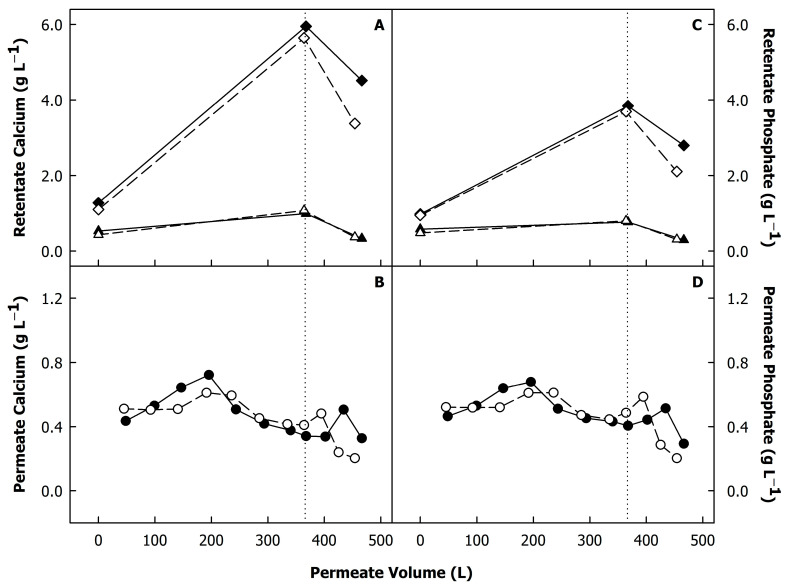
Total (diamonds) and soluble contents (triangles) of calcium (**A**) and phosphate (**C**) of skimmed milk and milk protein concentrates during microfiltration and diafiltration, and calcium (**B**) and phosphate (**D**) concentrations of the microfiltration permeates and different stages of the process. The dotted lines indicate the beginning of the diafiltration step. Transmembrane pressure during diafiltration was 0.05 MPa (closed symbols) or 0.10 MPa (open symbols).

Figure 4 summarises the concentrations of calcium and phosphate of the original skimmed milk, the retentates, and the corresponding centrifugal supernatants (Figure 4A,C), as well as in all permeate samples (Figure 4B,D). The total calcium and phosphate (Figure 4; diamonds) of the retentates increased during the concentration step in the same way as the protein content, and the amount of micellar calcium and phosphate remained constant at ~32 and ~20 mg per g casein, respectively, which is in agreement with previous reports [16,18]. The micellar calcium and phosphate remained constant during diafiltration at a TMP of 0.05 MPa, whereas, in the case of the higher TMP, the concentrations decreased to ~29 and 17 mg per g casein. The differences in total calcium and phosphate between a low and high TMP were significant (p < 0.05). This indicates that a higher TMP can cause some dissociation of micellar salts.

During the first half of the concentration step, the calcium and phosphate contents of the permeates were close to or higher than those of the serum phase of the skimmed milk (~0.48 g L^−1^ and ~0.53 g L^−1^, respectively), whereas they were previously reported to be lower due to interactions with non-permeable proteins such as non-micellar caseins or aggregated whey proteins [16,18,23]. This underlines the permeation of some casein micelles through the membrane, which would increase the calcium and phosphate contents of the permeates due to the colloidal calcium phosphate associated with the casein micelles. The diafiltration step decreased both total and soluble calcium and phosphate due to the milieu exchange, i.e., the permeation of soluble salts including those dissociated from the casein micelles.

**Figure 5:**
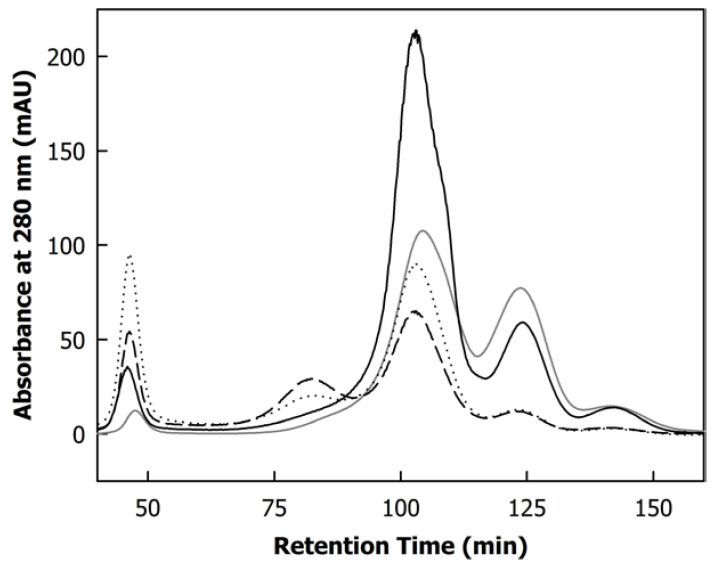
Size exclusion chromatograms of centrifugal supernatants of skimmed milk (grey line) and microfiltration retentate before diafiltration (black full line), after diafiltration at 0.05 MPa transmembrane pressure (black dashed line), and after diafiltration at 0.1 MPa transmembrane pressure (black dotted line). Curves are representative chromatograms.

Figure 5 shows the size exclusion chromatograms of the centrifugal supernatants of skimmed milk and the microfiltration retentates, showing the typical pattern of non-protein material eluting at <60 min and >120 min, caseins at 75–90 min, and native whey proteins at 90–120 min [20]. There was a clear trend towards an increase in the casein peak with diafiltration, as was also reported previously [16]. The presence of caseins in the centrifugal supernatant is consistent with a change in the serum phase’s ionic composition, causing dissociation of the casein micelles. A higher elution peak was found when the concentrates were prepared at a low TMP during diafiltration (0.05 MPa) compared to diafiltration at a TMP of 0.1 MPa. This result disputes the hypothesis that membrane filtration at a higher operating TMP causes more dissociation of casein due to the presence of high shear forces and a deposit layer on the membrane. It was concluded that a higher TMP, causing a greater compression of the deposit layer [8,11], will instead reduce the ratio of soluble to colloidal casein. This could be due to a higher amount of calcium present in the gel layer, as shown by the lower permeation of calcium in the permeates at a high TMP (Figure 4B). Interestingly, Weinberger et al. [10] reported that the amount of calcium bridges in the deposit layer increases with increasing pH of the substrate, which typically occurs during diafiltration with water [16,18]. On the other hand, the proportion of calcium bridges was lower at a higher TMP; however, the sum of stabilising bonds in the deposit layer increased with increasing TMP [10], indicating also a stronger attraction of proteins in the bulk phase. Therefore, the non-sedimentable casein aggregates formed during DF might become embedded in the deposit layer to a greater extent in case of a higher TMP.

It is also important to note that it has been previously shown that the concentration of milk proteins by membrane filtration causes a smaller release of caseins into the serum phase than using osmotic stressing, which is a gentle, osmotic pressure-driven concentration method carried out without shear [24]. In that case, it was hypothesised that the shear forces and transmembrane pressure during membrane filtration facilitates rearrangements of casein micelles, limiting their dissociation. Likewise, the dissociation of casein micelles might be limited when operating at a high TMP.

## 4. Conclusions

Skimmed milk was concentrated by microfiltration and subsequently diafiltered against soft water using a tangential cross-flow membrane. The effect of TMP on protein permeation was studied in relation to the formation of non-sedimentable casein aggregates in the retentates. The differences in the operating TMP conditions during diafiltration showed little effect on the whey protein permeation. On the contrary, non-sedimentable casein aggregates were formed to a greater extent at a lower TMP. It was concluded that the greater compression of the deposit layer and/or more pronounced rearrangements of the casein micelles in the bulk phase at a higher TMP limited the dissociation of caseins into the serum phase. Both non-sedimentable casein aggregates and modifications in the casein micelle structure due to rearrangements during membrane filtration might have considerable effects on the techno-functional properties of MCCs. The present study therefore contributes important knowledge to the understanding of the processing–structure–function interrelations of milk protein concentrates, which is crucial to predict the functionality of MCCs depending on their processing history.

## Data Availability

The data presented in this study are available on request from the corresponding author.
